# Donor Chimerism of B Cells and Nature Killer Cells Provides Useful Information to Predict Hematologic Relapse following Allogeneic Hematopoietic Stem Cell Transplantation

**DOI:** 10.1371/journal.pone.0133671

**Published:** 2015-07-30

**Authors:** Ying Jiang, Liping Wan, Youwen Qin, Xiaorui Wang, Shike Yan, Kuangcheng Xie, Chun Wang

**Affiliations:** 1 Department of Hematology, Shanghai First People’s Hospital, Medical College, Shanghai Jiaotong University, Shanghai, China; 2 Department of Central Laboratory, Shanghai First People’s Hospital, Medical College, Shanghai Jiaotong University, Shanghai, China; French Blood Institute, FRANCE

## Abstract

In this study we investigated the correlation between donor chimerism status and disease relapse following allogeneic hematopoietic stem cell transplantation (allo-HSCT). The chimerism of Fluorescence-activated cell sorter (FACS) sorted CD3+T lymphocytes of 153 cases, CD56+CD16+NK lymphocytes of 153 cases and CD19+B lymphocytes of 31 cases with acute B lymphoblastic leukemia (B-ALL) was analyzed post-transplant utilizing polymerase chain reaction amplification of short tandem repeats (PCR-STR). A total of 33 patients (33/153, 21.6%) had recurrent disease. The positive predictive values of declining donor chimerism for hematologic and isolated extramedullary relapse were 58.8% and 10% (P=0.018, Chi-Square). The positive predictive values of declining donor chimerism in BMB, BMT, BMNK and PBB for hematologic relapse were 11.6%, 0%, 0% and 0% under close monitoring in patients with B-ALL. Only the donor chimerism in BMB significantly decreased in the group with hematologic relapse as compared with the group without hematologic relapse (P=0.00, Independent-samples T test) in patients with B-ALL. The median drop of donor chimerism in PBT, BMT, PBNK and BMNK were 0%, 0%, 5.9% and 2.8% one or two weeks prior to hematologic relapse in patients with non-B-ALL. The donor chimerism in PBNK significantly decreased prior to hematologic relapse in the group with hematologic relapse as compared with the group without hematologic relapse (P=0.022, Independent-samples T test).These data suggest donor chimerism of BMB can be used to predict the occurrence of hematologic relapse in patients with B-ALL. Donor chimerism decrease in PBNKwas associated with a somewhat increased risk of hematologic relapse in patients with non-B-ALL. Therefore, our results reveal a more effective path to individually predict for hematologic relapse by dynamic monitoring different cell lineages in different disease.

## Introduction

Hematopoietic stem cell transplantation is now an established treatment for a number of non-malignant and malignant conditions [1,2,3]. However, the relapse rate is still an important problem for patients after allogeneic hematopoietic stem cell transplant (allo-HSCT) [4]. PCR-based analyses of short tandem repeats (STRs) are commonly used and are accurate and applicable to allogeneic transplant recipients [5,6]. The reoccurrence or increase in autologous hematopoiesis after allogeneic transplantation has been linked to incipient disease relapse [7]. So chimerism analysis offers the possibility of identifying recurrence of underlying disease [8,9].

The microsatelite analysis by PCR of chimerism within specific leukocyte subsets isolated from peripheral blood or bone marrow samples by flow-sorting techniques provides more specific information on assessment of impending complications at a significantly higher sensitivity [10]. Detection of chemerism of T cells has been recommended by several guidelines [11], but did not appear to work well for prediction of disease relapse [12]. Here we analyzed the quantitative chimerism kinetics of 153 consecutive patients who underwent allo-HSCT at Shanghai First People’s Hospital between November 2004 to July 2012. The follow-up period was from November 2004 to October 2013. We explore possibilities of prediction of disease relapse by detecting chimerism of subsets of lymphocytes.

## Materials and Methods

### Patients

One hundred and fifty three consecutive patients received allo-HSCT at Shanghai First People’s Hospital. The study has been approved by ethical committee of Shanghai First People’s Hospital, Medical College, Shanghai Jiaotong University. Written informed consents from the patients were obtained for the use of this sample in research. Patients characteristics are shown in Table 1. The source of stem cells was peripheral stem cells. No patient received lymphocytes infusions before their hematologic relapse or donor chimerism decrease.

**Table 1:** Patients characteristics.

Median age	33(6∼60)
gender (M/F)	91/62
Median follow up	11.2(0.2–91.5)months
Conditioning	
myeloblative conditioning	122
reduced intensity conditioning	31
Diagnosis	
AML	54
ALL	32
CML	39
MM	4
MDS	9
NHL or HL	13
Myelofibrosis	2
Donor type	
HLA-matched related donor	49
Haploidentical related donor	17
HLA-matched unrelated donor	45
HLA-mismatched unrelated donor	42
GVHD prophylaxis	
CSA/MMF/MTX/ Basiliximab	18
CSA/MMF/MTX/ATG	4
CSA/MMF/ Basiliximab	19
CSA/MMF/ATG	4
CSA/MMF/MTX	32
CSA/MMF	43
CSA/MTX	20
CSA	2
FK506/MMF/MTX/ Basiliximab	2
FK506/MMF/ Basiliximab	1
FK506/MMF/MTX	3
FK506/MTX	5

### Specimens

Peripheral blood from recipients and donor grafts were obtained before allo-HSCT. 4–10 ml peripheral blood (PB) or 2ml bone marrow (BM) from the recipients were collected at regular intervals of every two weeks until achieving complete donor chimerism (CDC) and then each month post-transplant, if the sample was available. If donor chimerism decrease of any patient was detected in any subset, the patient would be monitored every week till chimerism of all subsets reached CDC. PB and BM were collected into the EDTA tubes. Patients were systematically examined in parallel with the chimerism detection.

### Sorting subsets of lymphocytes

Peripheral blood mononuclear cells (PBMC) which extracted from PB or BM samples with ficoll stained for surface antigens. The antibodies were CD3+-PE, CD19+-FITC, CD56+CD16+-PE (BD). The CD3+ thymus derived cell (T cell) and CD56+CD16+ natural killer cell (NK cell) were sorted for all 153 patients by the Fluorescence-activated cell sorter (FACS) (FACSvantageSE). CD19+ bursa dependent lymphocyte (B cell) was sorted for 31 cases with acute B lymphoblastic leukemia (B-ALL) by the Fluorescence-activated cell sorter (FACS) (FACSvantageSE). The final quantity was 1*10^3^–1*10^6^ cells of each subset. The purity of the sorted cells was more than 96%. Two days was the maximum time between specimen collection and FACS sorting.

### Quantitative detection of PCR-STR

**Fig 1:**
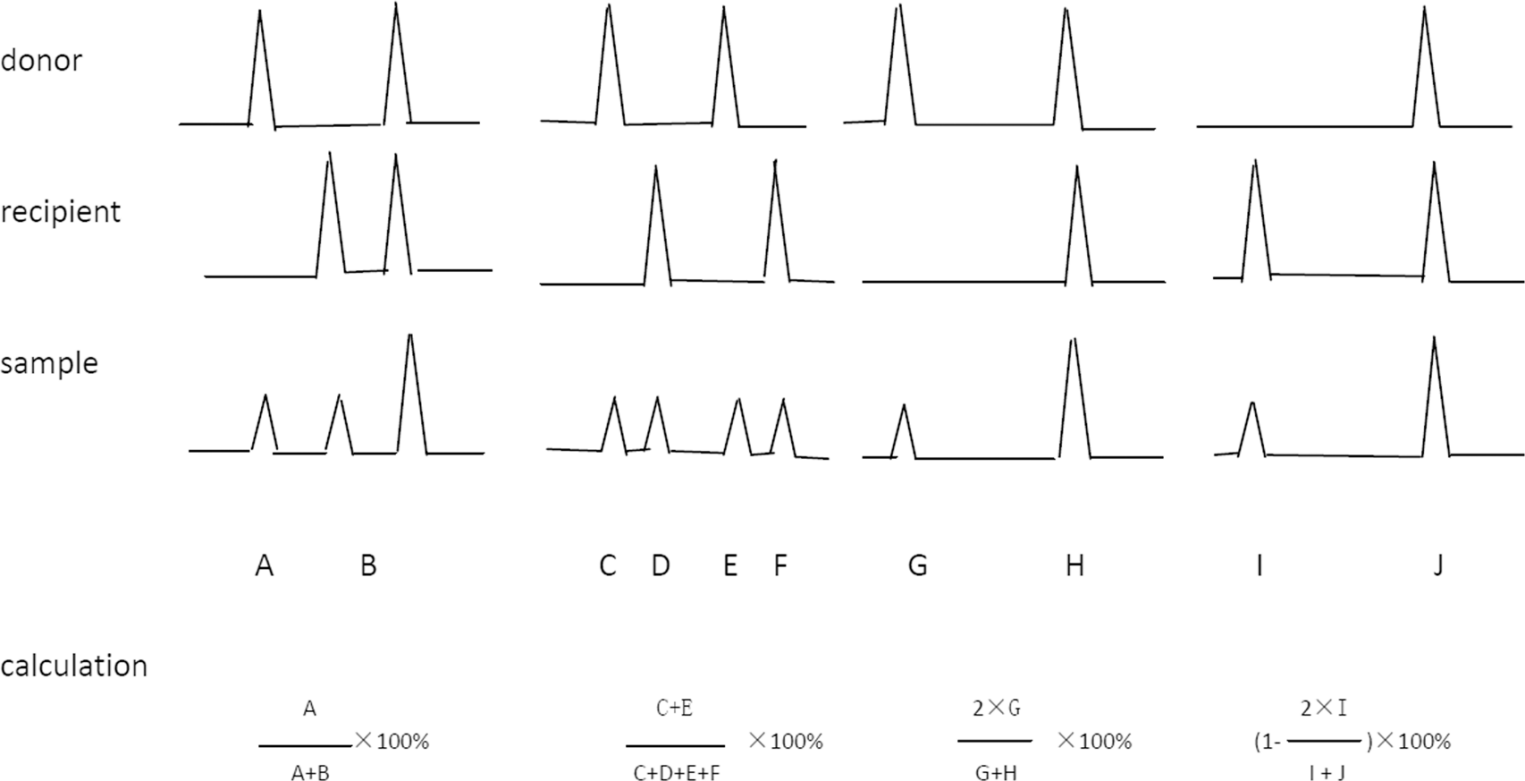
Calculation of peak area in chimerism. The first line is the samples of the donor. The second line is the samples of the recipient prior to transplantation. The third line is the samples examined following transplantation. The fourth line is the calculation formula of peak area in chimerism.

We did operate according to the protocols of commercial kits. Genomic DNA which isolated from all cell subsets sorted by FACS was extracted by ReadyAmp Genomic DNA Purification System Kit (Promega). The final concentration of DNA was 0.5ng/ul. One day was the maximum time between FACS sorting and DNA extraction. Amplifications were performed with the AmpFlSTR Profiler Plus Kit (Applied Biosystems, USA) on an ABI 7300 Fast Real-Time PCR System. All Multiplex PCR reactions were completed in 20μl volume. The volume/quantity of DNA was 5ul/2.5ng. Cycle conditions were as follows: 95°C for 11 minutes followed by 28 cycles of 94°C for 1 minute,58°C for 1 minute,72°C for 1 minute, and a final step consisting of 45 minutes at 60°C. The PCR products were analyzed using capillary electrophoresis on ABI PRISM 3130 Genetic Analyzer. The GeneMapper v4.0 provided a complete STR genotyping. Serial samples were analyzed for degrees of donor-recipient chimerism using PCR of informative minisatellite regions [13,14]. PCR products were detected on ten alleles: D3S1358、vWA、FGA、Amelogenin、D8S1179、D21S11、D18S51、D5S818、D13S317 and D7S820. The loci on ten alleles of every patient were detected in donor and recipient samples before transplantation. The informative loci were selected from which had the difference between donor and recipient. Calculation of peak area in chimerism is as follows (Fig 1).

### Definition

Disease relapse is defined as isolated donor chimerism decrease, hematologic and isolated extramedullary relapse. Hematologic relapse is according to the criteria of Williams Hematology. Isolated extramedullary relapse is meaning blasts at other sites outside of the bone marrow. Isolated donor chimerism decrease is diagnosed when the donor chimerism decreased without hematologic and extramedullary relapse. Complete donor chimerism (CDC) is 100% donor chimerism in all cell subsets of the sample which we examined.

### Statistics

Statistical analysis was performed using SPSS 13.0 software. T test and Chi-Square were used. P values <0.05 were considered as significant.

## Results

### Predicting ability of Chimerism status to isolated extramedullary relapse and hematologic relapse

Of all patients, 144 achieved sustained complete donor chimerism (CDC). There were 7 patients who experienced graft rejection. We didn’t find the correlation between the conditioning regimen and the graft rejection. The other two patients had died of Cerebral Hemorrhage before their chimerism reached CDC. Decreased donor chimerism was observed in samples of 73 patients. A total of 33 patients (33/153, 21.6%) had hematologic and isolated extramedullary relapse, reaching a cumulative incidence of hematologic relapse of 15% (23/153) and isolated extramedullary relapse of 6.5% (10/153) from November 2004 to October 2013.

Of the ten isolated extramedullary relapsed patients, one patient (No. 31) converted from CDC to mix chimerism (MC) in the BMT lineage with a drop of 10% one week prior to isolated extramedullary relapse, but no further decline was detected at the time of isolated extramedullary relapse. One patient (No. 33) was detected the drop of 11% and 8% in BMT and BMNK lineages at the moment of isolated extramedullary relapse. All of the remaining 8 cases sustained CDC in the whole study period (Table 2). The positive predictive value of declining donor chimerism for isolated extramedullary relapse was 10%.

**Table 2:** Relapsed patients characteristics.

No	gender	age	diagnosis	time of relapse	relapse	Donor chimerism decrease
1	M	47	CML	32D	H	Y
2	F	18	B-ALL	7.5M	H	Y
3	F	41	B-ALL	55D	H	Y
4	M	44	B-ALL	7M	H	Y
5	M	48	MM	9M	E	N
6	F	14	B-ALL	5.5M	H	Y
7	M	33	MM	2M	E	N
8	M	20	AML	12M	E	N
9	M	48	AML	12M	H	Y
10	F	16	B-ALL	18M	H	Y
11	F	54	B-ALL	3.6Y	H	Y
12	M	21	AML	5W	H	Y
13	F	20	B-ALL	9M/17M	H	Y
14	M	21	AML	8W	H	Y
15	F	33	AML	21W	H	Y
16	M	35	CML	2M	H	Y
17	M	30	AML	10M	H	Y
18	M	20	B-ALL	11M	H	Y
19	M	26	ALL	4M	H	Y
20	M	28	B-ALL	3.5M	E	N
21	M	17	AML	2M	H	Y
22	F	30	B-ALL	2M	H	Y
23	M	22	NHL	6W	H	Y
24	M	35	AML	1Y	E	N
25	M	53	B-ALL	3M	H	Y
26	F	32	B-ALL	14M/16M /20M	H	Y
27	M	18	CML	9M	H	Y
28	M	32	NHL	18M	E	N
29	M	55	AML	3M	E	N
30	F	39	NHL	6W	E	N
31	M	49	AML	5W	E	Y
32	M	34	B-ALL	7M	H	Y
33	M	21	NHL	4W	E	Y

M: male; F: female; H: hematologic relapse; E: isolated extramedullary relapse; Y: yes; N: no.

Decreased donor chimerism was detected in the samples of all of the 23 hematologic relapsed patients (100%). And there were 10 patients observed declining donor chimerism prior to the hematologic relapse in 17 patients under regular monitoring (10/17, 58.8%) except 6 patients (No. 4, 9, 10, 11, 25 and 32) without regular monitoring.

So the positive predictive values of declining donor chimerism for hematologic and isolated extramedullary relapse were 58.8% and 10% (P = 0.018, Chi-Square). The predict sensitivity of donor chimerism for hematologic relapse is higher than that for isolated extramedullary relapse.

### Predicting ability of Chimerism status of different lymphocyte subsets to B-ALL relapse

In 28 B-ALL patients, isolated donor chimerism decrease was detected in 3 patients and hematologic relapse occurred in 12 patients.

The median drop of donor chimerism in B, T and NK cells of bone marrow (BMB, BMT and BMNK) were 74.3%, 13.2% and 19.6% at the point of hematologic relapse. Donor chimerism in BMB significantly decreased as compared with those in BMT (P = 0.000, Paired-samples T tests) and BMNK (P = 0.002, Paired-samples T tests). The median levels of donor chimerism decreased in BMB, BMT and BMNK were 11.6%, 0% and 0% one or two weeks prior to hematologic relapse. The B cells, not T or NK cells, dropped as the earliest and deepest lineage of BM.

The median drop of donor chimerism in B cells of bone marrow and peripheral blood (BMB and PBB) were 74.3% and 7.2% at the moment of hematologic relapse and 11.6% and 0% one or two weeks prior to hematologic relapse. The donor chimerism in BMB is significantly different from the donor chimerism in PBB at the moment of hematologic relapse (P = 0.003, Paired-samples T tests) and at one or two weeks prior to hematologic relapse (P = 0.05, Paired-samples T tests). The drop of donor chimerism in BMB was earlier and deeper than that in PBB.

Of the 3 cases who underwent isolated donor chimerism decrease, the median drops of donor chimerism in BMB, BMT, BMNK and PBB were 0%, 1%, 0% and 0%. Only the donor chimerism in BMB significantly decreased in the group with hematologic relapse as compared with the group without hematologic relapse (P = 0.00, Independent-samples T test), while no significant difference was found in the donor chimerism in BMT, BMNK, PBB (P>0.05, Independent-samples T test).

As a result, the donor chimerism in BMB dropped as the earliest and deepest lineage in patients with B-ALL. The declining donor chimerism in BMB can be used as a sensitive marker to predict the hematologic relapse in patients with B-ALL. (Table 3)

**Table 3:** Characteristics of the B-ALL patients who experienced hematologic relapse.

No	Time point of relapse	Drop of donor chimerism before hematologic relapse	Drop of donor chimerism when hematologic relapse
2	7.5M	0(PBT/PBNK/PBB/BM)	BM 50.8%
3	55D	0(PBT/PBNK/PBB)	BM 75.5%
4	7M	*	BMT 10.7% PBT 0 BMNK 8.3% PBNK 0 BMB 58.1% PBB 0
6	5.5M	BM 11%	BM 86.8%
10	18M	*	BMT 76.4% PBT 56.3% BMNK82.5% PBNK 62.5% BMB 87.3% PBB 71%
11	3.6Y	*	BMT 0 BMNK76.9% BMB 64.8%
13	9M	*	BMT 33.3% PBT 0 BMNK 15% PBNK 0 BMB 78.5% PBB 7.2%
13	17M	BMB 56.6%	BMT 8% BMNK 0 BMB 78.5%
18	11M	BMT 3.7% BMNK 6.7% BMB 4.1%	BMT 44.6% PBT 0 BMNK56.8% PBNK 0 BMB 51.7% PBB 0
18	14M	BMB11.6%	BMT 12.1% PBT 0 BMNK15.7% PBNK 0 BMB 53.5% PBB 0
22	2M	BMB 22.6%	BMT 8% PBT 0 BMNK 8.6% PBNK 0 BMB 69.8% PBB 52.5%
25	3M	*	BMT 9.3% PBT 0 BMNK10.7% PBNK 0 BMB 74.3% PBB 52.5%
26	14M	*	BMT 76.4% BMNK90.2% BMB 100%
26	16M	BMB 3.1%	BMT 65.6% BMNK 92% BMB 100%
26	20M	BMB 28.3%	BMT 62.3% BMNK88.8% BMB 87.8%
32	7M	*	BMT 13.2% BMNK19.6% BMB 58.4%

data weren’t monitored BM: unfractionated cells of bone marrow.

### Predicting ability of Chimerism status of different lymphocyte subsets to non-B-ALL relapse

The B-cell donor chimerism showed having nothing to do with relapse in patients with non-B-ALL in our previous study, so only donor chimerism in T and NK cells were monitored in this study.

In the group with hematologic relapse, the median drop of donor chimerism in NK cells of peripheral blood (PBNK) was 14.9% at the point of hematologic relapse and 5.9% one or two weeks prior to hematologic relapse. The median drop of donor chimerism in PBNK was 3.2% in the group with isolated donor chimerism decrease. The donor chimerism in PBNK significantly decreased prior to hematologic relapse in the group with hematologic relapse as compared with the group without hematologic relapse (P = 0.022, Independent-samples T test).

In the group with hematologic relapse, the median drop of donor chimerism in NK cells of bone marrow (BMNK) was 50.85% at the point of hematologic relapse and 2.8% one or two weeks prior to hematologic relapse. The median drop of donor chimerism in BMNK was 1.97% in the group with isolated donor chimerism decrease. No significant difference was found in the donor chimerism decrease in BMNK prior to hematologic relapse in the group with hematologic relapse as compared with that in the group without hematologic relapse (P = 0.133, Independent-samples T test).

The median drop of donor chimerism in PBT and BMT were 1.35% and 16.15% at the point of hematologic relapse and 0% and 0% one or two weeks prior to hematologic relapse.

The drop of donor chimerism in PBNK and BMNK, not PBT or BMT, were detected prior to hematologic relapse. The declining PBNKdonor chimerism, can be used to predict the hematologic relapse in patients with non-B-ALL.

## Discussion

Allogeneic hematopoietic stem cell transplantation (allo-HSCT) is a curative treatment for many patients suffering from malignant and non-malignant hematological disorders. One of the most useful tools for monitoring is the assessment of hematopoietic chimerism which occurs after alloHSCT and describes the percentage of donor hematopoietic and lymphoid cells in a transplant recipient [15,16]. STR-PCR is defined as a gold standard method for quantificational monitoring donor chimerism by IBMTR [17]. The selection of sub-cellular population studied with high sensitive technology allows a rapid and efficient intervention before the onset of clinical signs in patient and could improve the patient's follow-up. Several reports [10,18–25] highlight the importance of sub-cellular population chimerism documentation enable to ascertain a stable engraftment and to detect early relapse. Our result showed that the hematologic relapse, not isolated extramedullary relapse, can be predicted by chimerism status. The declining donor chimerism in BMB and PBNK cells can be used to predict the hematologic relapse in patients with B-ALL and non-B-ALL, respectively.

Maeng H et al. [26] report a patient who showed an extramedullary relapse in her uterus, without bone marrow recurrence, while the examination of peripheral blood cells showed full donor chimerism. The positive predictive value of declining donor chimerism for isolated extramedullary relapse was also the lower one (10%) in our study. Ohashi H et al. [27] also suggest that the graft-versus-leukemia effects might not be as effective in the CNS as in the BM, even when complete T-lymphoid chimerism is achieved. So the observation of donor chimerism of the cases with isolated extramedullary relapse, without bone marrow recurrence, does not by itself constitute a risk factor for predicting relapse and should not be used to guide therapeutic intervention.

In recipients of allogeneic HSCT, identification of patients at high risk for hematologic relapse and use of interventions post-transplant to augment the graft-versus-tumor effect are possible areas of donor chimerism kinetics. Some investigations [8,15] evaluate that the results of chimerism monitoring confirm that the failure of achieving a CDC or missing from CDC can predict the hematologic relapse of the disease. Many studies focus on the prediction effect of the donor chimerism of some date following transplant, some are succeed [28,29], and some are failed [30]. In our previous study [20,23], it failed to predict for relapse such as day 28 after transplant. But the dynamic monitoring for chimerism status can individually predict the outcome of patients. Therefore, it is possible that hematologic relapse can be predicted earlier by chimerism status and prevented by some treatment. Then the problem is how to predict for hematologic relapse.

Actually it is useful in some disease to target selected sub-populations in order to have an earlier detection of hematologic relapse on cell fractions [25]. In our previous study [23], we found that although decreased donor chimerism was observed in all of patients with hematological relapse (100%), it was found to be ineffective to predict the occurrence of relapse by monitoring donor chimerism of the whole sample of PB and BM as compared with the subsets. In this study, the positive predictive value of declining donor chimerism in BMB, BMT, BMNK and PBB for hematologic relapse were 11.6%, 0%, 0% and 0% under close monitoring in patients with B-ALL. Only the donor chimerism in BMB significantly decreased in the group with hematologic relapse as compared with the group without hematologic relapse (P = 0.00, Independent-samples T test) in patients with B-ALL, while no significant difference was found in the donor chimerism in BMT, BMNK, PBB (P>0.05, Independent-samples T test) in patients with B-ALL. Hence, the observation of donor chimerism decrease of BMB can be used to predict the occurrence of hematologic relapse in patients with B-ALL.

Some studies suggest special cell subsets could predict for relapse, such as T-cell in ALL, AML and NHL [31], CD34 cell in MDS and AML [32]. In our previous study [23], data show the declining donor chimerism in B-cell could not predict for hematologic relapse in patients with non-B-ALL. In this study, although the frequency of declining donor chimerism of T-cell was more than that of NK-cell at the point of hematologic relapse, the median drop of donor chimerism in PBT and BMT were 0% and 0% one or two weeks prior to hematologic relapse. The declining donor chimerism in T-cell could not be used to predict the hematologic relapse in patients with non-B-ALL.

The median drop of donor chimerism in PBNK and BMNK were 5.9% and 2.8% one or two weeks prior to hematologic relapse. The donor chimerism in PBNK significantly decreased prior to hematologic relapse in the group with hematologic relapse as compared with the group without hematologic relapse (P = 0.022, Independent-samples T test). Donor chimerism decrease in PBNK was associated with a somewhat increased risk of hematologic disease recurrence.

In summary, decreasing donor chimerism of PBNK and BMB cells can be detected and predictive for hematologic relapse of patients with non-B-ALL and B-ALL by sequentially quantitative monitoring of their chimerism which sorted by FACS. Our results reveal a more effective path to individually predict for hematologic relapse by dynamic monitoring different cell lineages in different disease. Future studies are needed to address more samples to verify them.
